# Far-red triplet sensitized *Z*-to-*E* photoswitching of azobenzene in bioplastics†

**DOI:** 10.1039/d2sc04230d

**Published:** 2022-09-14

**Authors:** Pankaj Bharmoria, Shima Ghasemi, Fredrik Edhborg, Raúl Losantos, Zhihang Wang, Anders Mårtensson, Masa-aki Morikawa, Nobuo Kimizuka, Ümit İşci, Fabienne Dumoulin, Bo Albinsson, Kasper Moth-Poulsen

**Affiliations:** Department of Chemistry and Chemical Engineering, Chalmers University of Technology Kemivägen 4 412 96 Gothenburg Sweden pankajbharmoria@gmail.com kasper.moth-poulsen@chalmers.se; Department of Chemistry and Molecular Biology, University of Gothenburg Kemigården 10, Göteborg 412 96 Gothenburg Sweden; The Institute of Materials Science of Barcelona, ICMAB-CSIC Bellaterra 08193 Barcelona Spain; Catalan Institution for Research & Advanced Studies, ICREA Pg. Lluís Companys 23 Barcelona Spain; Université de Paris Cité and CNRS, ITODYS F-75006 Paris France; Universidad de La Rioja, Departamento de Química, Centro de Investigación en Síntesis Química Madre de Dios, 53 26006 Logroño Spain; Department of Applied Chemistry, Graduate School of Engineering, Center for Molecular Systems (CMS) Kyushu University 744 Moto-oka, Nishi-ku Fukuoka 819-0395 Japan; Gebze Technical University, Chemistry Department 41400 Gebze Kocaeli Turkey; Acıbadem Mehmet Ali Aydınlar University, Faculty of Engineering and Natural Sciences, Biomedical Engineering Department Ataşehir Istanbul Turkey

## Abstract

We report the first example of direct far-red triplet sensitized molecular photoswitching in a condensed phase wherein a liquid azobenzene derivative (Azo1) co-assembled within a liquid surfactant–protein film undergoes triplet sensitized *Z*-to-*E* photoswitching upon far-red/red light excitation in air. The role of triplet sensitization in photoswitching has been confirmed by quenching of sensitizer phosphorescence by *Z*-Azo1 and temperature-dependent photoswitching experiments. Herein, we demonstrate new biosustainable fabrication designs to address key challenges in solid-state photoswitching, effectively mitigating chromophore aggregation and requirement of high energy excitations by dispersing the photoswitch in the trapped liquid inside the solid framework and by shifting the action spectrum from blue-green light (450–560 nm) to the far-red/red light (740/640 nm) region.

## Abstract 2

We report the first example of direct far-red endothermic triplet sensitized *Z*-to-*E* photoswitching of azobenzene derivative (Azo1) in a condensed phase of a liquid Azo1 co-assembled within a liquid surfactant-protein bioplastic film in air.

## Graphical abstract

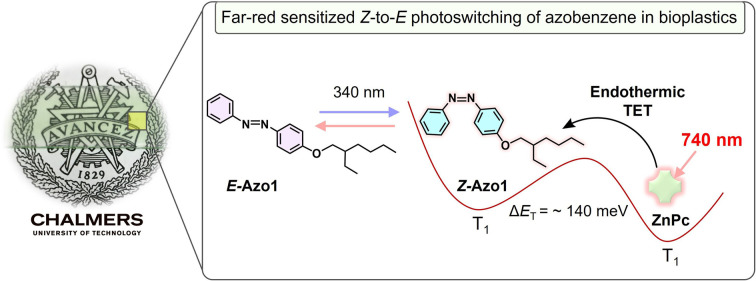

## 1 Introduction

Molecular photoswitches are organic molecules that undergo geometrical isomerization upon irradiation with light at their absorption wavelengths.^1–4^ Due to the spatiotemporal control of the two isomers with distinct polarity and optical properties, photoswitches have attracted a plethora of photonic applications, such as photon energy storage,^5–8^ photo-actuation,^9,10^ light-activated drug release,^11^ nano-imprint lithography,^10^ opto-spintronics, *etc.*^12^ This is because the light modulated properties of isomers can be further imparted into the bulk properties of matrix materials, if the application is sought in the solid state.^13–16^ Among the known photoswitches (azobenzene, thioindigo, hydrazone, diarylethene, spiropyran, norbornadiene, dihydroazulene, *etc.*), azobenzene based photoswitches are among the most popular due to their chemical stability and structural tunability.^4^ The parent *E*-azobenzene shows a strong π–π* transition at around 320 nm and a weak n–π* transition at 440 nm, whereas the *Z*-azobenzene shows a strong n–π* transition at 440 nm and a weaker π–π* transition at 280 nm.^17^ While photoisomerization of the azobenzene chromophore is usually fast in the solution, it is difficult to achieve in the solid state. This is because chromophores stack in high density causing a steric hindrance, less free space, and concomitant low orientational entropy.^18–20^ Although some reported azobenzene derivatives exhibit *E*–*Z* photoisomerization in the solid state,^8,21–24^ the low penetration depth of high absorption energies (UV-Vis light) further limits the photoisomerization efficiency due to competing absorption between the isomers, especially if the application is sought in thick materials.^25,26^

**Fig. 1:**
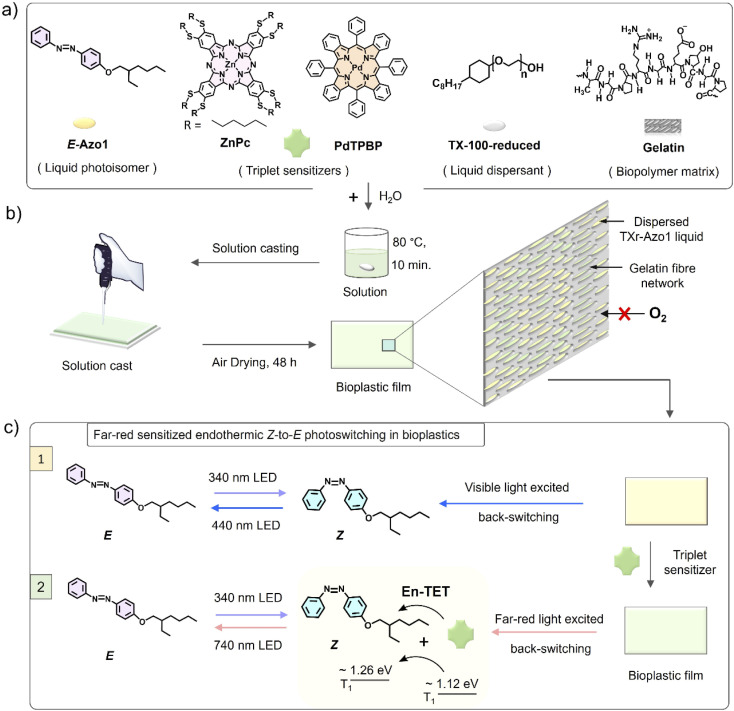
(a) Molecular structures of *E*-Azo1, ZnPc, PdTPBP, TX-100-reduced and gelatin, (b) schematic of the preparation of solid G-TXr-Azo1 or G-TXr-Azo1-ZnPc or G-TXr-Azo1-PdTPBP photoswitching bioplastic films and film structure, and (c) illustration of the *Z*-to-*E* photoswitching of Azo1 in the absence and presence of a far-red sensitizer in bioplastics. En-TET = endothermic triplet energy transfer.

To facilitate efficient solid-state photoswitching of azobenzene, it is generally required to increase the free volume and rotational freedom. For example, various strategies like covalent functionalization of small molecules and polymers,^8,27–32^ template functionalization on carbon nanotubes,^33^ nanoparticles,^34,35^ and nano-cages,^36^ and surface anchoring on semiconductors^37–39^ have been developed to achieve efficient solid-state photoswitching. These strategies have contributed significantly towards realizing solid-state photoswitching applications of azobenzene.^13–16^ However, further advancements in simple switching matrices, supporting high molecular densities with free rotation, along with the shift in photoswitching wavelengths beyond the absorption of the isomers and towards the low energy and more penetrable far-red/NIR region are highly desired. In this direction, red/NIR upconverted photons by transition metal doped upconversion nanoparticles (UCNPs)^40–42^ and triplet–triplet annihilation upconversion (TTA-UC)^43^ materials have been used for photoswitching of dithienylethene,^40^ azobenzene-modified poly(acrylic acid) copolymers,^41^ and azotolane^42,43^ both in solution and condensed states.^40–43^ The photoswitching energy of azobenzene can also be red-shifted, either by synthesizing a new direct low energy absorbing derivative^44^ or through indirect low energy excited photoswitching of azobenzene *via* direct triplet sensitization,^45^ as recently demonstrated by the Durandin research group in a DMSO solution with an oxygen scavenger.^46^ The photoswitching *via* direct triplet sensitization is different from UCNPs/TTA-UC photon-assisted photoswitching. Because it involves direct non-radiative triplet energy transfer from sensitizer triplets to the photoswitch triplets for inducing photoswitching. Herein, we introduce two facile approaches that together can enable efficient photoswitching of azobenzene derivatives in the condensed phase: (1) a bioplastic matrix approach that enables enough structural flexibility for efficient solid-state photo-switching and (2) direct endothermic triplet sensitization that extends the optical action spectrum of photoswitching into the low energy, far-red/red regions. Photoswitching bioplastics were simply prepared by air drying of an aqueous solution of a liquid azobenzene derivative (Azo1),^7,47^ co-assembled within a liquid surfactant, Triton X-100-reduced (TXr), and the protein gelatin (G) as shown in Fig. 1a and b. The Azo1-TXr liquid dispersed inside the semicrystalline gelatin film has a liquid-like environment with sufficient space for free molecular rotation around the double bond for photoisomerization in the bioplastics.

To shift the action spectrum of Azo1 photoswitching towards low energy far-red/red light, we doped the G-TXr-Azo1 films with micromolal concentrations of either a far-red sensitizer octa (hexylthio) Zinc(ii) phthalocyanine (ZnPc) or a red sensitizer Pd(ii) *meso*-tetraphenyl tetrabenzoporphine (PdTPBP) as shown in Fig. 1a. The G-TXr-Azo1-ZnPc and G-TXr-Azo1-PdTPBP bioplastics showed efficient triplet sensitized *Z*-to-*E* photoswitching upon excitation with 740 nm and 640 nm LED light, thanks to the oxygen blocking ability of gelatin which protected chromophore triplets from oxygen quenching.^48,49^ The triplet energy of ZnPc (1.12 eV)^50^ is lower than that of *Z*-Azobenzene (∼1.26 eV).^51^ Hence, this is the first example of direct far-red sensitized solid-state photoswitching *via* endothermic triplet energy transfer (Δ*E*_T_ = ∼+0.14 eV). Contrary to this, the PdTPBP sensitized *Z*-to-*E* photoswitching occurs *via* exothermic triplet energy transfer (Δ*E*_T_ = ∼−0.3 eV) due to the higher triplet energy of PdTPBP (1.55 eV) compared to that of *Z*-azobenzene (∼1.26 eV).^51^ It is to be mentioned here that we have used experimental triplet energy of non-derivatized *Z*-azobenzene^51^ for Azo1, since the Azo1 did not show any phosphorescence even at 77 K. However, for a valid comparison we calculated the triplet energy of Azo1 and non-derivatized azobenzene over singlet optimized geometries using the B3LYP/cc-pVTZ method.^52^ The triplet energy was computed using the restricted open formalism to improve the comparability between singlet and triplet energies (see Method section, ESI†). No significant differences were found between the triplet energy of non-derivatized *Z*-azobenzene (1.875 eV) and *Z*-Azo1 (1.86 eV) as can be seen in Table S1, ESI.† The calculated triplet energy of non-derivatized azobenzene is comparable to the reported values calculated using different DFT functionals (Table S2†) which confirms the accuracy of these calculations.

From these results, we assume that the experimental triplet energy of *Z*-Azo1 should not change significantly when compared to non-derivatized *Z*-azobenzene (1.26 eV).^51^ Hence the assumption that endothermic triplet energy transfer occurred from ZnPc to *Z*-Azo1 should remain valid.

The biopolymer matrix offers a key advantage of biodegradation over petroleum-derived polymers as a bulk host matrix for photonic application to avoid post-utility disposal issues.^53,54^ In addition to biodegradation, the nano-heterogenous polar structure of biopolymers^55^ supports chromophore dispersion, and their thick fiber network can block oxygen in the case of oxygen-sensitive photochemical reactions.^48,49^ Previous efforts on azobenzene fabrication in biopolymers are limited to electrospinning of the covalent functionalized Azo-cellulose-azobenzene film.^32^ However, this film did not show any photoswitching.^32^ Therefore, this work is the first example of an efficient molecular photoswitching bioplastic. The molecular structures of Azo1, TXr, Zn-Pc, PdTPBP and gelatin are shown in Fig. 1a, and a representative schematic of film preparation and photoswitching is shown in Fig. 1b and c.

## 2 Results and discussion

### 2.1. Preparation and characterization of photoswitching films

The G-TXr-Azo1, G-TXr-Azo1-PdTPBP, and G-TXr-Azo1-ZnPc films were prepared by simple mixing of their aqueous solutions at 80 °C, followed by drop casting and air drying at room temperature for 48 h (Fig. 1b). Details of the film preparation are given in the Experimental section of ESI.† The final concentrations of the chromophores in the air-dried films are: Azo1 = 1.64 mmol kg^−1^, PdTPBP = 82 μmol kg^−1^ and ZnPc = 82 μmol kg^−1^ and those of gelatin and TXr are 89.4% and 10.5% respectively. The air-dried G-TXr-Azo1, G-TXr-Azo1-PdTPBP, and G-TXr-Azo1-ZnPc films are shown in Fig. 4a, 5a, and 6a. We carried out detailed structural characterization of the G-TXr-Azo1-ZnPc film using scanning electron microscopy (SEM), powder X-ray diffraction (P-XRD), and differential scanning calorimetry (DSC).

**Fig. 2:**
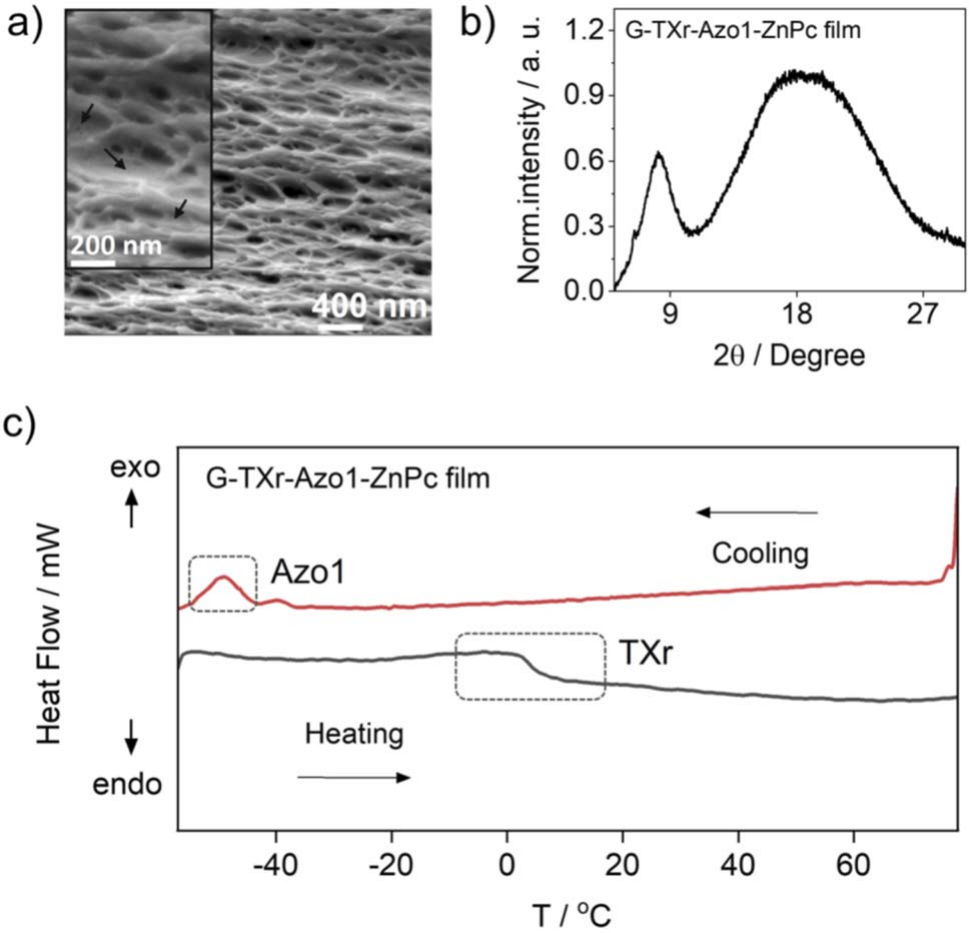
Structural characterization of the G-TXr-Azo1-ZnPc film, (a) cross-sectional SEM image (inset shows a zoomed image indicating gelatin fibers plasticized by dispersed liquid indicated by arrows), (b) P-XRD pattern, and (c) DSC thermogram.

The cross-sectional SEM image of the G-TXr-Azo1-ZnPC film showed a porous network of thick fibers of the gelatin containing a plasticizing liquid that could be the dispersed TXr-Azo1 (Fig. 2a). The P-XRD diffraction pattern of the G-TXr-Azo1-ZnPc film confirmed its semicrystalline nature from the small peak at 2*θ* = 8.2°, corresponding to the crystalline triple helices of gelatin with an inter-helix distance of 1.1 nm (Fig. 2b).^48,56^ The liquidity of dispersed Azo1-TXr inside the semicrystalline gelatin film was confirmed from the endothermic glass transition of TXr at ∼3 °C and the exothermic crystallization peak of Azo1 at −50 °C from the DSC thermogram (Fig. 2c and S1, ESI†). The glass transition of TXr in the film does not show a sharp endothermic peak as in native TXr liquid (Fig. S1, ESI†). This could be due to the plasticization of TXr, confined in the film at low temperature. Such behaviour is previously reported for TX-100 trapped in mesoporous polymers.^57^ However, at room temperature, TXr remains as a trapped liquid to foster molecular diffusion of chromophores.^48^

**Fig. 3:**
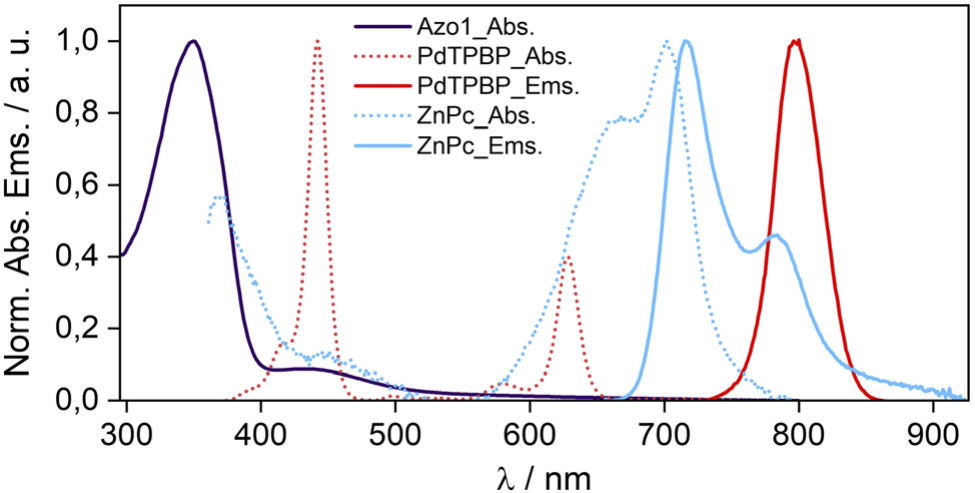
Absorption and emission spectra of *E*-Azo1, PdTPBP, and ZnPc in the G-TXr film.

To confirm the molecular dispersion of chromophores in G-TXr films we measured their separate absorption and emission spectra (Fig. 3). The absorption spectrum of *E*-Azo1 shows peaks due to the strong π–π* transition at 347 nm and the weak n–π* transition at 434 nm.^17^ The absorption spectrum of PdTPBP shows a Soret band at 442 nm and Q bands at 580 and 627 nm, and the phosphorescence maximum at 796 nm (1.55 eV). The similarity of these peaks to those of Azo1 and PdTPBP dissolved in toluene indicates their molecular dispersion in the G-TXr film. The absorption spectrum of ZnPc shows an 11 nm blue shift of the Q band from 713 nm in toluene (Fig. S2, ESI†) to 702 nm in the G-TXr-ZnPc film along with a new peak at 667 nm and spectral broadening due to H-aggregation (Fig. 3, cyan dots).^58,59^

The fluorescence spectrum of ZnPc in the G-TXr-ZnPc film shows peaks at 715 and 784 nm, which are also blue-shifted by 6 and 9 nm respectively compared to that in toluene. Such spectral changes in phthalocyanines are well known due to the formation of photo-emissive H-aggregates in solution and solid states.^58,59^ The phosphorescence maximum of ZnPc in the G-TXr-ZnPc film was observed at 1107 nm (1.12 eV, Fig. S3, ESI†),^50^ which is 0.14 eV lower than the triplet energy of *Z*-azobenzene (1.26 eV).^51^ However, ZnPc still transfers triplet energy to *Z*-Azo1 endothermically to induce *Z*-Azo1 to *E*-Azo1 photoswitching in the G-TXr-Azo1-ZnPc bioplastic film discussed in the subsequent section.

### 2.2. Photoswitching in the G-TX-Azo1-bioplastic film

**Fig. 4:**
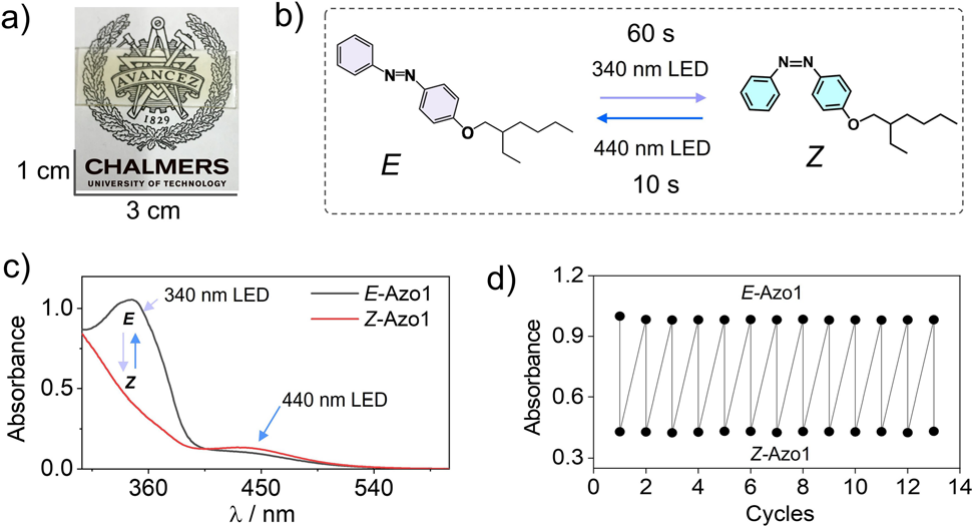
(a) Digital image of the semi-transparent G-TXr-Azo1 film, (b) Illustration of *E*-Azo1 ↔ *Z*-Azo1 reversible photoswitching upon 340 and 440 nm LED excitation of the film, (c) absorption spectra of *E*-Azo1 and *Z*-Azo1 in the film, and (d) cyclic photoswitching of *E*-Azo1 and *Z*-Azo1 in the film upon LED excitation at 340 nm.

First, we measured the feasibility of Azo1 photoswitching in the G-TXr-Azo1 film without triplet sensitizers (Fig. 4a). In the dark the Azo1 exists as an *E*-Azo1 isomer with strong absorption peaks at 347 nm, and 434 nm respectively (Fig. 4c).

Upon excitation with a 340 nm LED (power density = 2.8 mW cm^2^) for 60 s the film showed *E*-Azo1 to *Z*-Azo1 photoswitching with a sharp decrease in absorbance at 347 nm and a small rise at 434 nm. The excitation of the G-TXr-*Z*-Azo1 film with a 440 nm LED (power density = 27.6 mW cm^−2^) resulted in complete *Z*-Azo1 → *E*-Azo1 back photoswitching within 10 s (Fig. 4b and c). For these measurements the G-TXr-Azo1 film was excited at a 90° angle with respect to the LED (Fig. S4a and b, ESI†). The rate constant of *E* to *Z* photoisomerization in the film (*k*_*E* to *Z*_ = 0.0045 s^−1^, discussed later in the kinetics section of the current MS) is comparable to that of Azo1 dissolved in TXr (*k*_*E* to *Z*_ = 0.012 s^−1^, Fig. S5, ESI†). This indicates no significant suppression of Azo1 photoisomerization in the dispersed TXr phase of the bioplastic film. Hence, the G-TXr-Azo1 film addresses the key issue of suppressed photoisomerization due to chromophore aggregation in the solid-state molecular photoswitching. The durability of photoswitching in the G-TXr-Azo1 film was further confirmed by measuring photoswitching for 13 consecutive cycles (Fig. 4d and S6, ESI†) with no measurable degradation.

### 2.3. Triplet sensitized photoswitching in the G-TX-Azo1-sensitizer bioplastic films

**Fig. 5:**
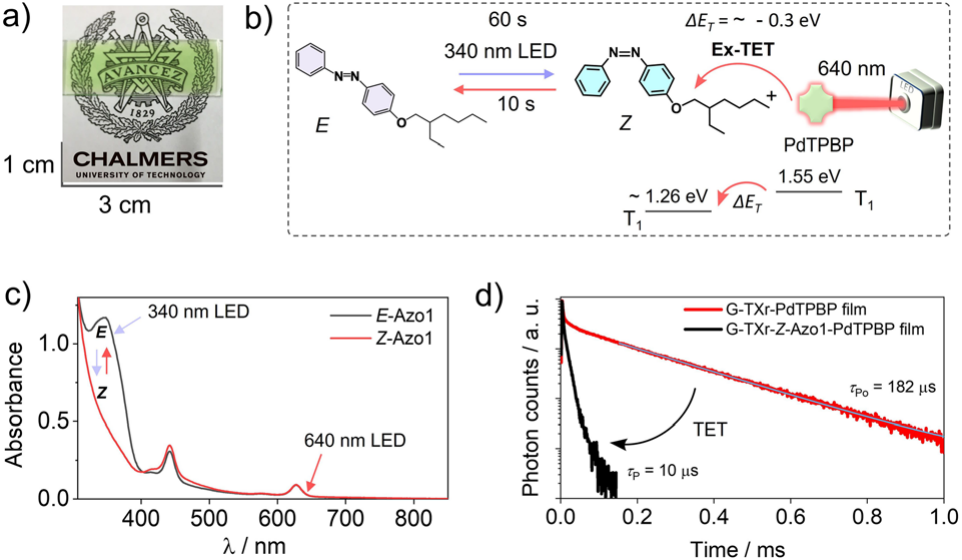
(a) Digital image of the semi-transparent G-TXr-Azo1-PdTPBP film, (b) illustration of triplet sensitized *Z*-Azo1 → *E*-Azo1 exothermic photoswitching upon 640 nm LED excitation of the film at a 90° angle, (c) absorption spectra of *E*-Azo1 and *Z*-Azo1 and PdTPBP in the film, and (d) phosphorescence decay profiles of G-TXr-PdTPBP and G-TXr-PdTPBP-*Z*-Azo1 films showing exothermic triplet energy transfer (*λ*_ex_ = 640 nm, and *λ*_em_ = 800 nm).

To shift the action spectrum of azobenzene photoswitching to low-energy excitations we used the direct triplet sensitization approach^45,46^ by doping G-TX-Azo1 films with far-red/red sensitizers. The triplet energy of an unsubstituted *Z*-azobenzene is 1.26 eV.^51^ As a red sensitizer, we used PdTPBP, having a triplet energy of 1.55 eV in the film (Fig. 3, solid red line). The approximate triplet energy gap between PdTPBP and *Z*-Azo1 is Δ*E*_T_ = ∼ −0.3 eV, which is suitable for exothermic triplet energy transfer to *Z*-Azo1 to induce photoswitching. Therefore, the prepared G-TXr-Azo1-PdTPBP film (Fig. 5a) was subjected to 90° excitation with 340 nm (power density = 2.8 mW cm^−2^) and 640 nm LEDs (power density = 14.1 mW cm^−2^), to induce reversible photoswitching (Fig. 5b).

The G-TXr-Azo1-PdTPBP film showed forward switching to the *Z*-Azo1 isomer in 60 s, whereas triplet sensitized back-switching to the *E*-Azo1 isomer occurred in 10 s (Fig. 5a) as observed from their absorption spectra (Fig. 5c). The triplet energy transfer from PdTPBP to *Z*-Azo1 in the film was confirmed from the decrease in the phosphorescence lifetime (Fig. 5d) and phosphorescence emission (Fig. S7, ESI†) of PdTPBP in the presence of Azo1 in the film. The efficiency of triplet energy transfer was measured by the quantum yield of triplet energy transfer (*Φ*_TET_) using phosphorescence lifetimes of PdTPBP in the absence (*τ*_Po_) and presence (*τ*_P_) of Azo1 in the film using eqn (1).1
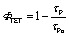


A high *Φ*_TET_ = 94% in the film indicates efficient triplet energy transfer from PdTPBP to *Z*-Azo1 to induce photoswitching. It is to be mentioned here that a long phosphorescence lifetime of PdTPBP observed in the absence of *Z*-Azo1 (*τ*_Po_ = 182 μs) also indicates protection of active triplets from quenching by molecular oxygen in the film. This is due to the thick fiber network of gelatin which blocks the entry of oxygen into the chromophore region in the G-TXr-*Z*-Azo1-PdTPBP film.^48^ For a detailed mechanism of the triplet protection from oxygen by gelatin fibers, please refer to our previous papers on gelatin–surfactant–chromophore systems.^48,49^ Furthermore, the durability of photoswitching in the G-TXr-Azo1-PdTPBP film was confirmed by measuring photoswitching for 13 consecutive cycles (Fig. S8, ESI†).

**Fig. 6:**
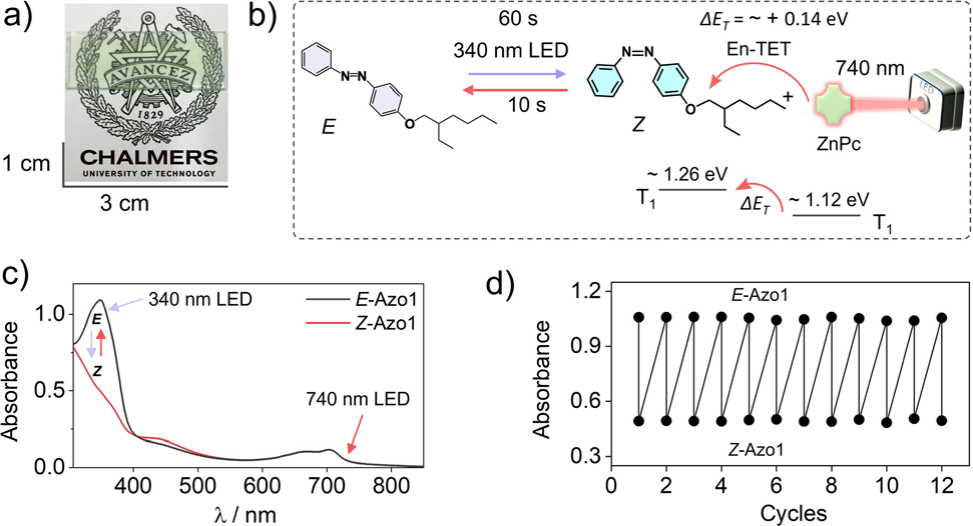
(a) Digital image of the semi-transparent G-TXr-Azo1-ZnPc film, (b) illustration of triplet sensitized *Z*-Azo1 → *E*-Azo1 endothermic photoswitching upon 740 nm LED excitation of the film at a 90° angle, (c) absorption spectra of *E*-Azo1 and *Z*-Azo1 and ZnPc in the film, and (d) cyclic photoswitching of *E*-Azo1 and *Z*-Azo1 states in the film upon LED excitation at 340 nm. En-TET = endothermic triplet energy transfer.

To further red-shift the action spectrum of the photoswitching of *Z*-Azo1 we used ZnPc as a far-red sensitizer. The ZnPc shows the phosphorescence maximum at 1107 nm, which corresponds to *T*_1_ = 1.12 eV^50^ (Fig. S3, ESI†), and hence has Δ*E*_T_ = ∼+0.14 eV with respect to the *T*_1_ = 1.26 of *Z*-azobenzene. Therefore, triplet energy transfer must happen endothermically^60–62^ to induce photoswitching.^46^ Similar to the red sensitized film, the G-TXr-Azo1-ZnPc film (Fig. 6a) showed forward photoswitching to *Z*-Azo1 upon excitation with a 340 nm LED (power density = 2.8 mW cm^−2^) in 60 s (Fig. 6b and c). We tested the feasibility of endothermic triplet energy between ZnPc and *Z*-Azo1 upon excitation of the G-TXr-*Z*-Azo1-ZnPc film with a 740 nm LED (power density = 17.1 mW cm^−2^). Interestingly, the film showed *Z*-Azo1 to *E*-Azo1 isomerization within 10 s despite Δ*E*_T_ = ∼+0.14 eV at 90° excitation (Fig. 6b and c). Hence, we demonstrated the photoswitching operation *via* an endothermic triplet energy transfer mechanism.

This energy deficit could be compensated by the higher concentration of the acceptor^46,60–62^ in the condensed liquid phase of the film. Moreover, the sufficient thermal energy at room temperature can also compensate the Δ*E*_T_ = ∼+0.14 eV for TET from ZnPc to *Z*-Azo1.^63,64^ Due to the weak phosphorescence of ZnPc at room temperature we could not calculate the efficiency of triplet energy transfer from ZnPc to *Z*-Azo1. However, the fast *Z*-Azo1 to *E*-Azo1 back-switching gives indirect evidence of the efficient endothermic triplet energy transfer. Also, Durandin *et al.* reported the feasibility of endothermic TET for photoswitching of azobenzene derivatives in a DMSO solution,^46^ which indeed is supportive evidence to our results in the condensed phase. Moreover, the shift in the action spectrum of photoswitching toward a more penetrating far-red region gives new directions to realize low energy excitation based efficient solid-state photoswitching systems. Furthermore, the durability of photoswitching in the G-TXr-Azo1-ZnPc film was confirmed by measuring photoswitching for 12 consecutive cycles (Fig. 6d and S9, ESI†).

### 2.4. Kinetics of isomerization in bioplastic films

Photoisomerization kinetics of all films were measured by recording their time-dependent absorption spectra at different excitation powers of LEDs (Fig. S10–S15, ESI†). For kinetics studies, films were excited at a 75° angle with respect to LEDs (Fig. S10a, S12a, S14a, ESI†). The rate constant of photoisomerization (*k*) was calculated using eqn (2).^47^2
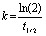
where *t*_1/2_ is the half-life of photoisomerization. The *t*_1/2_ was calculated by fitting the absorbance *vs.* time plots (Fig. S11a, c, S13a, c, S15a and c†) using a single exponential decay equation.

The rate constant of photoisomerization in all the films increased with an increase in excitation power for both *E* to *Z* and *Z* to *E* photoisomerization (see Fig. S11b, d, S13b, d, S15b, d and Tables S3–S5, ESI†).

**Fig. 7:**
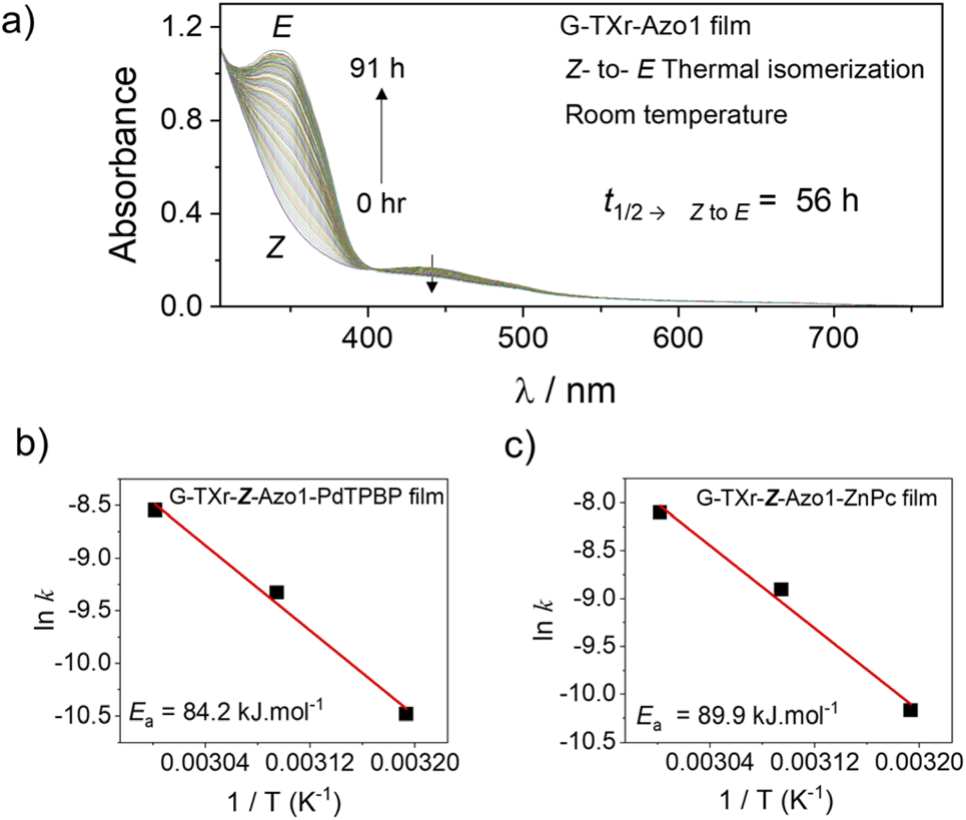
(a) Absorption spectrum of the G-TXr-*Z*-Azo1 film with time at room temperature showing *Z* to *E* isomerization. (b and c) Arrhenius plots (rate constant *vs.* temp.) of G-TXr-*Z*-Azo1-PdTPBP and G-TXr-*Z*-Azo1-ZnPc films respectively at 313, 323 and 333 K.

The *Z*-state of Azo1 is a metastable state which can also thermally back isomerize to the thermodynamically stable *E*-state. Hence to confirm that *Z* to *E* isomerization in the films is indeed due to the triplet sensitization rather than thermal, we measured the kinetics of *Z* to *E* isomerization of films at different temperatures. First, we measured the *Z* to *E* isomerization of the G-TXr-*Z*-Azo1 film without a sensitizer at room temperature (Fig. 7a and S161, ESI†).

At room temperature, the G-TXr-*Z*-Azo1 film shows a long *t*_1/2 → *Z* to *E*_ value of 56 h (Fig. S16, ESI†) with a small *k*_*Z* to *E*_ value of 3.4 × 10^−6^ s^−1^. The *t*_1/2 → *Z* to *E*_ value of 56 h is much longer than the *t*_1/2 → *Z* to *E*_ value of 8.4 s observed upon 440 nm LED excitation (Fig. S11c, ESI,† blue symbol) which indicates negligible thermal contribution towards *Z* to *E* photoisomerization.

To further confirm it we calculated the thermal activation barrier of *Z* to *E* isomerization in both G-TXr-*Z*-Azo1-PdTPBP and G-TXr-*Z*-Azo1-ZnPc films by measuring the back conversion at 313 K, 323 K, and 333 K using Arrhenius eqn (3).^47^3
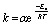
where *k* is the rate constant of isomerization, *α* is a pre-exponential factor, *T* is temperature and *R* is the gas constant in J K^−1^ mol^−1^. Finally, *E*_a_ refers to the activation energy of the *Z* to *E* isomerization in J mol^−1^, which corresponds to the thermal activation energy barrier.

For thermal measurements, we cut a piece of the film corresponding to the diameter of the quartz cuvette and fixed it so that it experiences uniform temperature (Fig. S17, ESI†). The absorption spectra, absorbance *vs.* time plots, and Arrhenius plots of G-TXr-*Z*-Azo1-PdTPBP and G-TXr-*Z*-Azo1-ZnPc films at 313, 323, and 333 K are shown in Fig. S18, S19, ESI† and Fig. 7b and c. Long *t*_1/2 → *Z* to *E*_ values of 3563 s and 2286 s were observed even at 333 K for G-TXr-*Z*-Azo1-PdTPBP and G-TXr-*Z*-Azo1-ZnPc films (Fig. S18f and S19f†), and they are much longer than the *t*_1/2 → *Z* to *E*_ values of 2.8 s and 5.4 s observed from 640 and 740 nm LED excitations (Fig. S13c and S15c†). This is due to the high thermal activation barrier of *Z* to *E* isomerization of Azo1 in these films, indicated by *E*_a_ = 84.15 kJ mol^−1^ for G-TXr-*Z*-Azo1-PdTPBP and *E*_a_ = 89.88 kJ mol^−1^ for G-TXr-*Z*-Azo1-ZnPc films (Fig. 7b and c). Therefore, such a high thermal activation barrier negates the possibility of a significant thermal contribution in the photoconversion experiments and indicates that direct triplet sensitization is indeed the main mechanism of *Z* to *E* photoisomerization in G-TXr-*Z*-Azo1-PdTPBP and G-TXr-*Z*-Azo1-ZnPc films.

Finally, it is to be mentioned here that the LED lights used have a rather broad emission spectrum (Fig. S20, ESI†). Also, the absorption spectrum of Azo1 (Fig. 2 and S2, ESI†) has a tail that ends near 620 nm. Therefore, the back-switching in the G-TXr-Azo1-PdTPBP film could be contributed by direct excitation rather than triplet sensitization. To shed this doubt, we measured the photoswitching of the G-TXr-Azo1 film upon continuous excitation with both 640 and 740 nm LEDs for 30 s at 90° excitation and did not observe any *Z*-Azo1 to *E*-Azo1 backswitching. Hence, it further confirms that back-switching in the sensitizer doped films is indeed due to the triplet energy transfer.

### 2.5. Durability of photoswitching in the bioplastic films

The durability of photoswitching in the bioplastic films was measured by measuring the reversible photoswitching of 9 month-old films stored at room temperature (Fig. S21, ESI†). All films showed similar photoswitching behaviours as shown before 9 months upon different LED excitations, which indicates that the films are very durable.

## 3 Conclusions

We have addressed key challenges in solid-state photoswitching of azobenzene like: (1) suppressed photoisomerization due to chromophore aggregation and (2) low energy excitation-based photoswitching, by developing a far-red/red sensitized azobenzene bioplastic film. These challenges were overcome by molecular dispersion of the azobenzene derivative, Azo1 along with a micromolal concentration of far-red/red triplet sensitizers in the viscous surfactant liquid trapped in the semicrystalline gelatin film. The liquid surfactant trapped in the solid-state provided a spacious hydrophobic fluidic environment for the molecular diffusion and photoisomerization of Azo1 for multiple cycles. In addition, the doping of the G-TXr-Azo1 film with triplet sensitizers allowed shifting of the action spectrum of the photoswitching of azobenzene toward the low energy far-red/red region *via* direct triplet sensitization of the *Z*-Azo1 isomer. Interestingly, far-red sensitized *Z*-Azo1 to *E*-Azo1 photoswitching occurred *via* an endothermic triplet energy transfer from ZnPc to *Z*-Azo1. This is the first example of an efficient molecular photoswitching bioplastic and the first example of direct far-red triplet sensitized photoswitching of azobenzene dispersed in the condensed liquid phase in the film *via* an endothermic triplet energy transfer. Moreover, the developed bioplastics technology gives new biosustainable platforms to fabricate solid-state photoswitching materials for energy harvesting applications.

## Data availability

Experimental data is available by email with corresponding authors and computational data is available by email with Rául Losantos.

## Author contributions

Pankaj Bharmoria and Kasper Moth-Poulsen conceptualized the idea of this work. Pankaj Bharmoria led the experimental work, supported by Shima Ghasemi, Fredrik Edhborg, Zhihang Wang, and Anders Mårtensson. Pankaj Bharmoria and Kasper Moth-Poulsen wrote and revised the manuscript supported by Fredrik Edhborg, Bo Albinsson and Nobuo Kimizuka. Masa-aki Morikawa and Nobuo Kimizuka lab synthesized Azo1 and Fabienne Dumoulin and Ümit İşci lab-synthesized ZnPc compounds respectively. Rául Losantos did DFT Calculations.

## Conflicts of interest

There are no conflicts to declare.

## Supplementary Material

SC-013-D2SC04230D-s001
