# Supplementary material for: Far-red triplet sensitized Z-to-E photoswitching of azobenzene in bioplastics
Source: Chem Sci. 2022 Sep 14;13(40):11904–11. doi: 10.1039/d2sc04230d (PMC9580493; doi:10.1039/d2sc04230d)
Supplement: SC-013-D2SC04230D-s001 [file SC-013-D2SC04230D-s001.pdf]

Electronic Supplementary Material (ESI) for Chemical Science.
This journal is © The Royal Society of Chemistry 2022

Supporting Information

Far-Red Triplet Sensitized *Z*- to - *E* Photoswitching of Azobenzene in Bioplastics

Pankaj Bharmoria,*[a,b] Shima Ghasemi,[a] Fredrik Edhborg,[a] Raúl Losantos,[e,f] Zhihang Wang,[a] Anders Mårtensson,[a] Masa-aki Morikawa,[g] Nobuo Kimizuka,[g] Umit Isci,[h] Fabienne Dumoulin,[i] Bo Albinsson,[a] Kasper Moth-Poulsen*[a,c,d]

[a]Department of Chemistry and Chemical Engineering, Chalmers University of Technology, Kemivägen 4, 412 96, Gothenburg, Sweden.

[b]Department of Chemistry and Molecular Biology, University of Gothenburg, Kemigården 10, 412 96 Göteborg, Gothenburg, Sweden.

[c]The Institute of Materials Science of Barcelona, ICMAB-CSIC, 08193, Bellaterra. Barcelona, Spain

[d]Catalan Institution for Research & Advanced Studies, ICREA, Pg. Lluís Companys 23, Barcelona, Spain

[e]Université de Paris Cité and CNRS, ITODYS, F-75006 Paris, France

[f]Universidad de La Rioja, Departamento de Química, Centro de Investigación en Síntesis Química, Madre de Dios, 53, 26006 Logroño, Spain

[g]Department of Applied Chemistry, Graduate School of Engineering, Center for Molecular Systems (CMS), Kyushu University 744 Moto-oka, Nishi-ku, Fukuoka 819-0395, Japan

[h]Gebze Technical University, Chemistry Department, 41400 Gebze Kocaeli, Turkey

[i] Acıbadem Mehmet Ali Aydınlar University, Faculty of Engineering and Natural Sciences, Biomedical Engineering Department, Ataşehir, Istanbul, Turkey

Experimental Section

Materials.

All the solvents and reagents were used as received. All solvents used in this work were purchased from Fischer Scientific. Palladium (II) meso-tetraphenyl porphine (PdTPBP) was purchased from Frontier Scientific. TX-100-reduced, and gelatin type A porcine skin (80 to 120 g bloom) were purchased from Sigma Aldrich. The azobenzene derivative (Azo1) was synthesized by Kimizuka Lab at Kyushu University, Japan). The synthesis and characterization of Azo1 in our previous publications.[1] Octa (hexylthio) Zinc (II) phthalocyanine (ZnPc) was synthesized according to the reported procedure.[2]

Methods.

Preparation of G-TXr-Azo1 film. 11.83 µl of Azo1 (42.258 mM in toluene), was taken in a glass vial and evaporated under reduced pressure. To this added 0.026 g of TX100-reduced (TXr), followed by stirring until Azo1 dissolves in TXr. To the resulting solution added 1 ml of MQ water, followed by stirring. Into the Azo1-TXr-H_2O solution added 0.22 g of gelatin type A (G), followed by stirring at 80 °C for 10 min. The hot sol was allowed to rest at room temperature for 2 minutes, followed by drop-casting of the 260 µl of the sol on a 3 x 1 cm glass plate and air drying for 48 h. The air-dried G-TXr-Azo1 film contain TX-r = 10.5%, gelatin = 89.4%, and Azo1 = 1.64 mmol.kg^{-1}. The light-yellow semi-transparent film obtained after air drying for 48 h is shown in Fig. 4a of the main manuscript.

Preparation of G-TXr-Azo1-PdTPBP film. 11.83 µl of Azo1 (42.258 mM in toluene), and 38 µl of PdTPBP (656 µM in toluene) were taken in a glass vial and evaporated under reduced pressure. To this added 0.026 g of TX100-reduced (TXr), followed by stirring until Azo1, and PdTPBP gets dissolved in TXr. To the resulting solution added 1 ml of MQ water, followed by stirring. Into the Azo1-TXr-H_2O solution added 0.22 g of gelatin type A (G), followed by stirring at 80 °C for 10 min. The hot sol was allowed to rest at room temperature for 2 minutes, followed by drop-casting of the 260 µl of the sol on a 3 x 1 cm glass plate and air drying for 48 h. The air-dried G-TXr-Azo1 film contain TX-r = 10.5%, gelatin = 89.4%, PdTPBP = 82 µmol.kg^{-1}and Azo1 = 1.64 mmol.kg^{-1}. The greenish semi-transparent film obtained after air drying for 48 h is shown in Fig. 5a of the main manuscript.

Preparation of G-TXr-Azo1-ZnPc film. 11.83 µl of Azo1 (42.258 mM in toluene), and 125 µl of ZnPc (200 µM in toluene) were taken in a glass vial and evaporated under reduced pressure. To this added 0.026 g of TX100-reduced (TXr), followed by stirring until Azo1 and ZnPc gets dissolved in TXr. To the resulting solution added 1 ml of MQ water, followed by stirring. Into the Azo1-TXr-H_2O solution added 0.22 g of gelatin type A (G), followed by stirring at 80 °C for 10 min. The hot sol was allowed to rest at room temperature for 2 minutes, followed by drop-casting of the 260 µl of the sol on a 3 x 1 cm glass plate and air drying for 48 h. The air-dried G-TXr-Azo1 film contain TX-r = 10.5%, gelatin = 89.4%, ZnPc = 82 µmol.kg^{-1}and Azo1 = 1.64 mmol.kg^{-1}. The light green semi-transparent film obtained after air drying for 48 h is shown in Fig. 6a of the main manuscript.

Optical Measurements. All UV–vis absorption spectra were recorded on a Varian Cary 50 spectrophotometers. Steady-state emission spectra were recorded on a Varian Eclipse spectrophotometer and FLS1000 Photoluminescence Spectrometer, Edinburgh instrument. The UV-Vis and emission spectra in solution were recorded using 2 mm pathlength quartz cuvettes. The phosphorescence spectra of PdTPBP in the presence and absence of Azo1 in the G-TXr film to measure triplet energy transfer were obtained using a home-built setup consisting of Coherent OBIS LS 633 nm diode laser as the excitation source, a 1681 SPEX monochromator, and a photomultiplier tube (PMT) detector. A 633 nm notch filter was used in front of the monochromator to reduce the scattered excitation light reaching the detector. The phosphorescence lifetimes of PdTPBP in the G-TXr-PdTPBP and G-TXr-Azo1-PdTPBP films were measured using Varian Eclipse 1 fluorescence spectrophotometer at λ_{ex} = 640 nm, and λ_{em} = 800 nm. The 340 nm and 440 nm LED's from THORLABS, whereas 640 nm and 740 nm LED's from LEDENGIN were used for photoswitching experiments. The LED powers were used were varied for different photoswitching experiments. For 90 ° excitation of different films the excitation power density (P_{ex}) of different LED's (P_{ex} = 2.8 mW cm^{-2} of 340 nm, 27.6 mW cm^{-2} of 440 nm, 14.1 mW cm^{-2} of 640 nm and 17.1 mW cm^{-2} of 740 nm) were used. For kinetics experiments at 75° excitation of different film samples three different LED powers of each LED mentioned

in the figures text were used. For photo-kinetics experiments both sample and LED's were covered with a black cloth in a dark room to avoid the interference of room light. For thermal isomerization measurements the films were cut in way so as they fit in the diameter of the quartz cuvette to experience uniform temperature. The phosphorescence spectrum of ZnPc was measured at 100 K, on Spex Fluorolog 3 spectrofluorometer (Horiba Jobin Yvon) equipped with a Hamamatsu H10330-45 NIR PMT detector at excitation wavelength of 700 nm.

Other Measurements. The X-ray powder diffraction of G-TXr-Azo1-ZnPc film was recorded using D8 Discover, Bruker Bragg-Brentano with Cu source. The differential scanning calorimetry (DSC) traces were obtained by using a TGA / DSC 3+ STARe system (METTLER TOLEDO) under N_2 atmosphere. Before performing measurements, samples were completely dried under vacuum for at least 24 h. The scanning rate was 10 °C min^{-1}. Reproducible thermogram of 3rd and 4th cycles were considered for presentation. The cross-section Scanning electron microscopy (SEM) images of the G-TXr-Azo1-ZnPc film were obtained using JEOL JSM-7800F Prime FEG SEM. The acceleration voltage was set to 10 kV and a secondary electron detector was used. Before SEM observation, the sample was dried under vacuum for two days and coated with gold using Edwards S150B gold sputter. The cross-section was obtained by breaking the film in half with hands.

Calculation of the Triplet Energy of Azo1. Over singlet optimized geometries using the B3LYP/cc-pVTZ method, the triplet energy was computed using the restricted open formalism to improve the comparability between singlet and triplet energies as described in Gagliardi, L., Orlandi, G., Bernardi, F. et al.[3] Additionally, the effect of the solvent environment was estimated using the iefpcm model for toluene, without big changes in the relative energies.

No significant differences were found between the Z-isomer of non-derivatized azobenzene and Z-Azo1 as can be seen in Table S1, for that we can assume that the triplet energy of Z-Azo1 is slightly lower (almost equivalent) than the one found for azobenzene, even being so close to the estimated method uncertainty. In contrast a more noticeable difference can be seen for *E*-isomer which is slightly higher than free azobenzene.

Table S1. Computed triplet energies at equilibrium singlet geometries using B3LYP/cc-pVTZ.

Compound	Azo1	Free azobenzene
E-isomer in vacuum	2.073 eV.	2.011 eV.
***Z*-isomer in vacuum**	**1.840 eV.**	**1.850 eV.**
E-isomer in toluene	2.076 eV.	2.011 eV.
***Z*-isomer in toluene**	**1.860 eV.**	**1.875 eV.**

Also, some functional benchmark was done to ensure a proper reproduction of the system by B3LYP observing, as described in the literature, a good description of the considered energies. Observing the typical shifts when changing the HF exchange percentage over the different functionals.

Table S2. Benchmark on free azobenzene with different DFT functionals. (Energies in eV. Referred to singlet minimum in each case)

E isomer	
B-LYP	1.886
B3LYP	1.982
CAM-B3LYP	2.020
wB97XD	2.036
PBE0	1.946
M062X	2.120
M06HF	2.356

Z isomer	
B-LYP	1.733
B3LYP	1.850
CAM-B3LYP	1.907
wB97XD	1.956
PBE0	1.842
M062X	2.041
M06HF	2.224

Supporting Figures

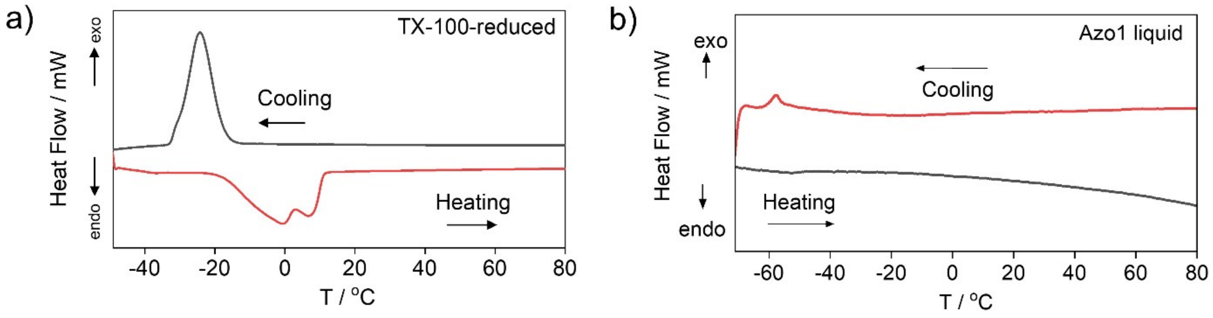

Fig. S1. DSC thermograms of vacuum dried a) TX-100-reduced, and b) Azo1 liquid. Scanning rate 10 ^{o}C min^{-1}.

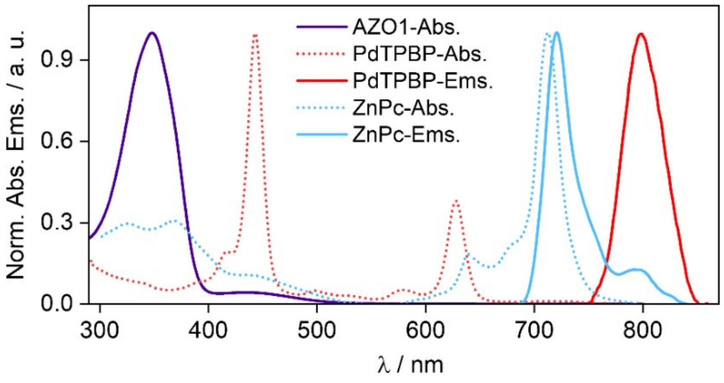

Fig. S2. Absorption, and emission spectra of Azo1, PdTPBP, and ZnPc dissolved in toluene

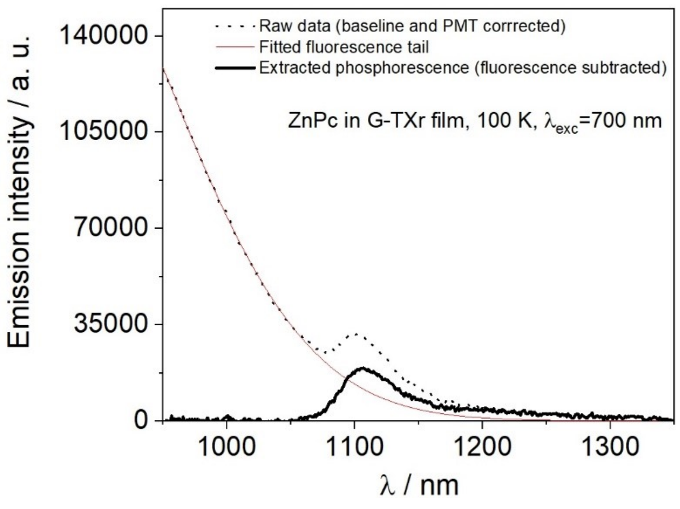

Fig. S3. The steady-state emission spectrum of G-TXr-ZnPc film at 100 K (dashed line, excitation wavelength 700 nm). The phosphorescence emission is seen as a small band around 1100 nm. The broad emission increasing at a lower wavelength is assumed to be the tail of the ZnPc fluorescence. Subtracting the fluorescence by fitting the emission tail up to 1065 nm to a gaussian curve (red line), the phosphorescence band can be isolated (solid black line), which has an emission maximum at 1107 nm.

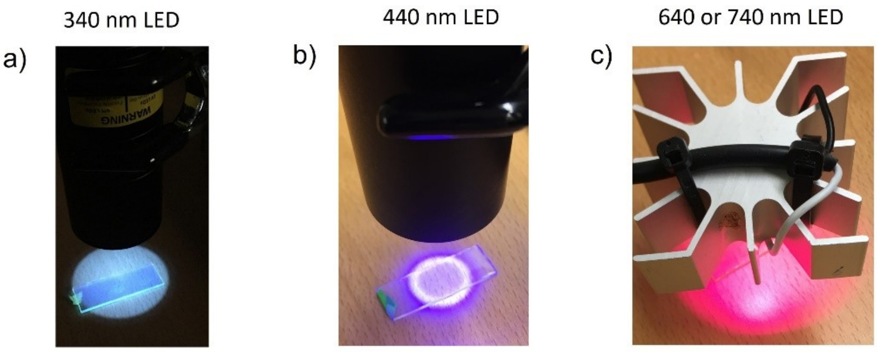

Fig. S4. Digital images of 90° angle excitation of photoswitching films with various LEDs. a) 340 nm LED, b) 440 nm LED, and c) 640 or 740 nm LED.

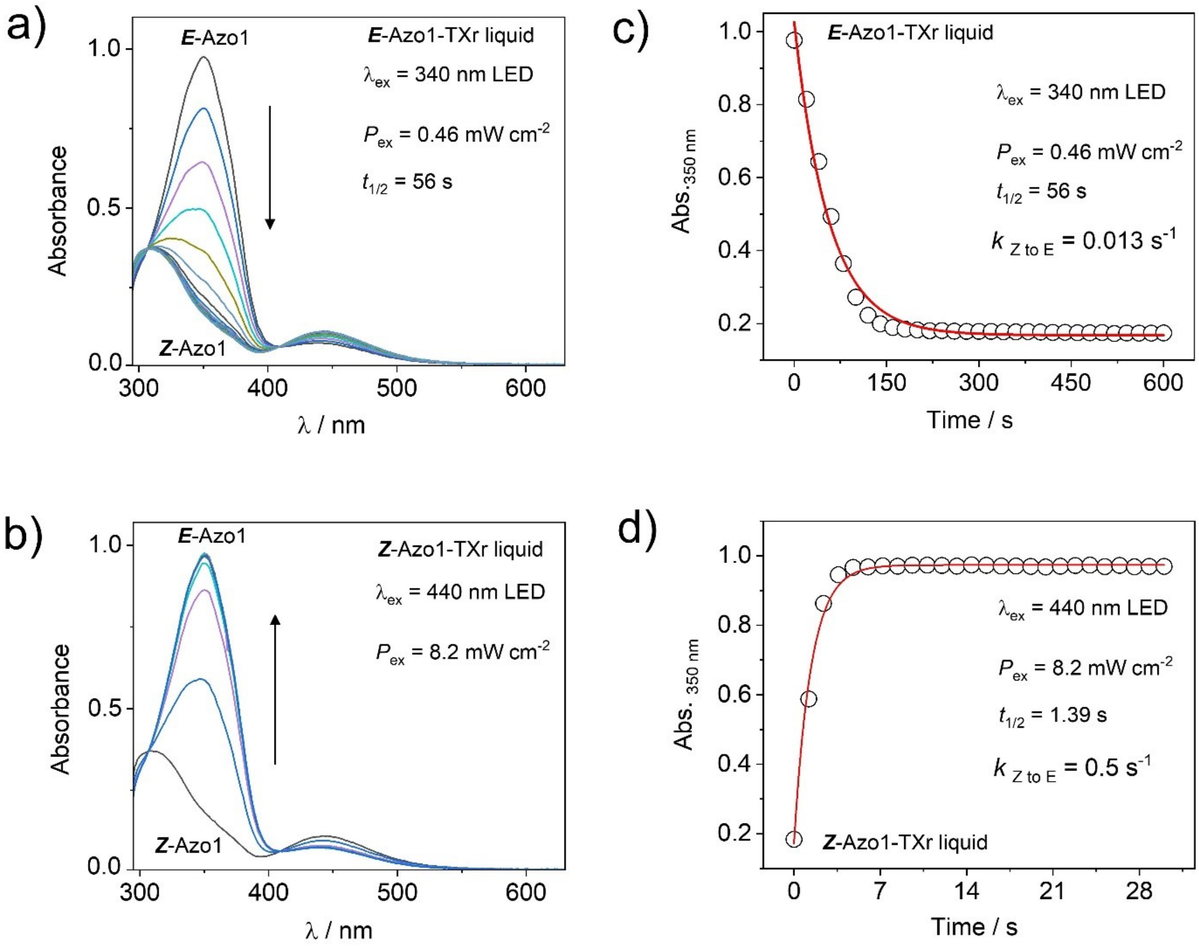

Fig. S5. Kinetics of the photoisomerization of Azo1 dissolved in TXr liquid. a, b) time-dependent absorption spectra of Azo1-TXr upon excitation with 340 nm and 440 nm LED, and c-d) absorbance vs. time profile of Azo1-TXr upon G-TXr-*Z*-Azo1 film upon excitation with 340 nm and 440 nm LED. Azo1 = 500 μM, cuvette pathlength = 0.1 cm, excitation angle 75°.

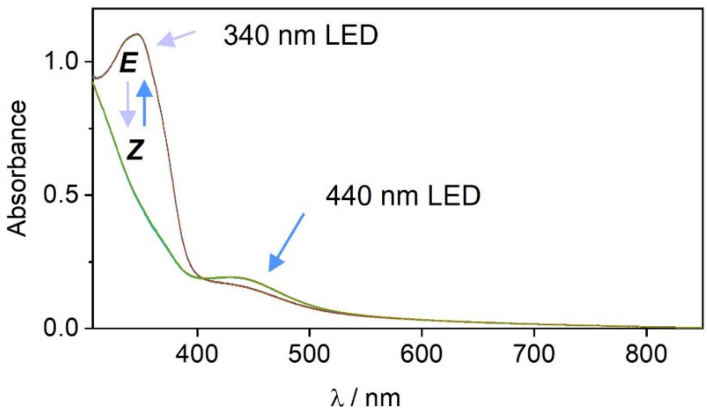

Fig. S6. Absorption spectra of *E*-Azo1 and *Z*-Azo1 for 13 consecutive cycles in G-TXr-Azo1 bioplastic film at 340 nm (P_{ex} = 2.8 mW cm^{-2}) and 440 nm (P_{ex} = 27.6 mW cm^{-2}) LED excitation.

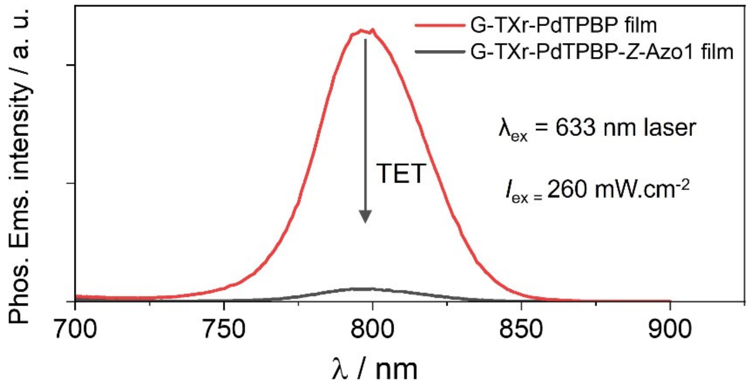

Fig. S7. Phosphorescence emission spectra of G-TXr-PdTPBP and G-TXr-PdTPBO-Azo1 films in air. The decrease in phosphorescence indicating triplet energy transfer (TET) from PdTPBP to *Z*-Azo1 in the film.

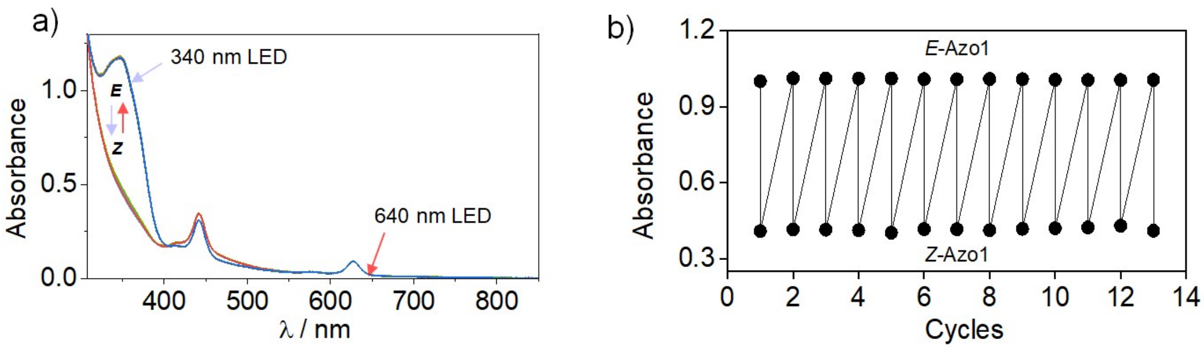

Fig. S8. a) Absorption spectra of *E*-Azo1 and *Z*-Azo1 for 13 consecutive cycles in G-TXr-Azo1-PdTPBP bioplastic film, and b) presentation of cyclic photoswitching between *E*-Azo1 and *Z*-Azo1 upon 340 (P_{ex} = 2.8 mW cm^{-2}) and 640 nm (P_{ex} = 14.1 mW cm^{-2}) LED excitation for 13 cycles.

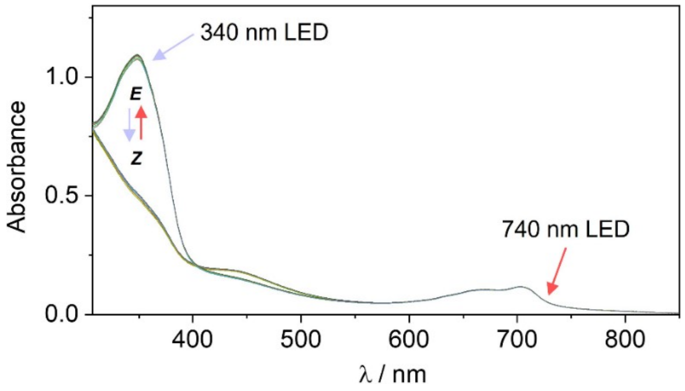

Fig. S9. Absorption spectra of *E*-Azo1 and *Z*-Azo1 for 12 consecutive cycles in G-TXr-Azo1-ZnPc bioplastic film upon 340 (P_{ex} = 2.8 mW cm^{-2}) and 740 nm (P_{ex} = 17.1 mW cm^{-2}) LED excitation.

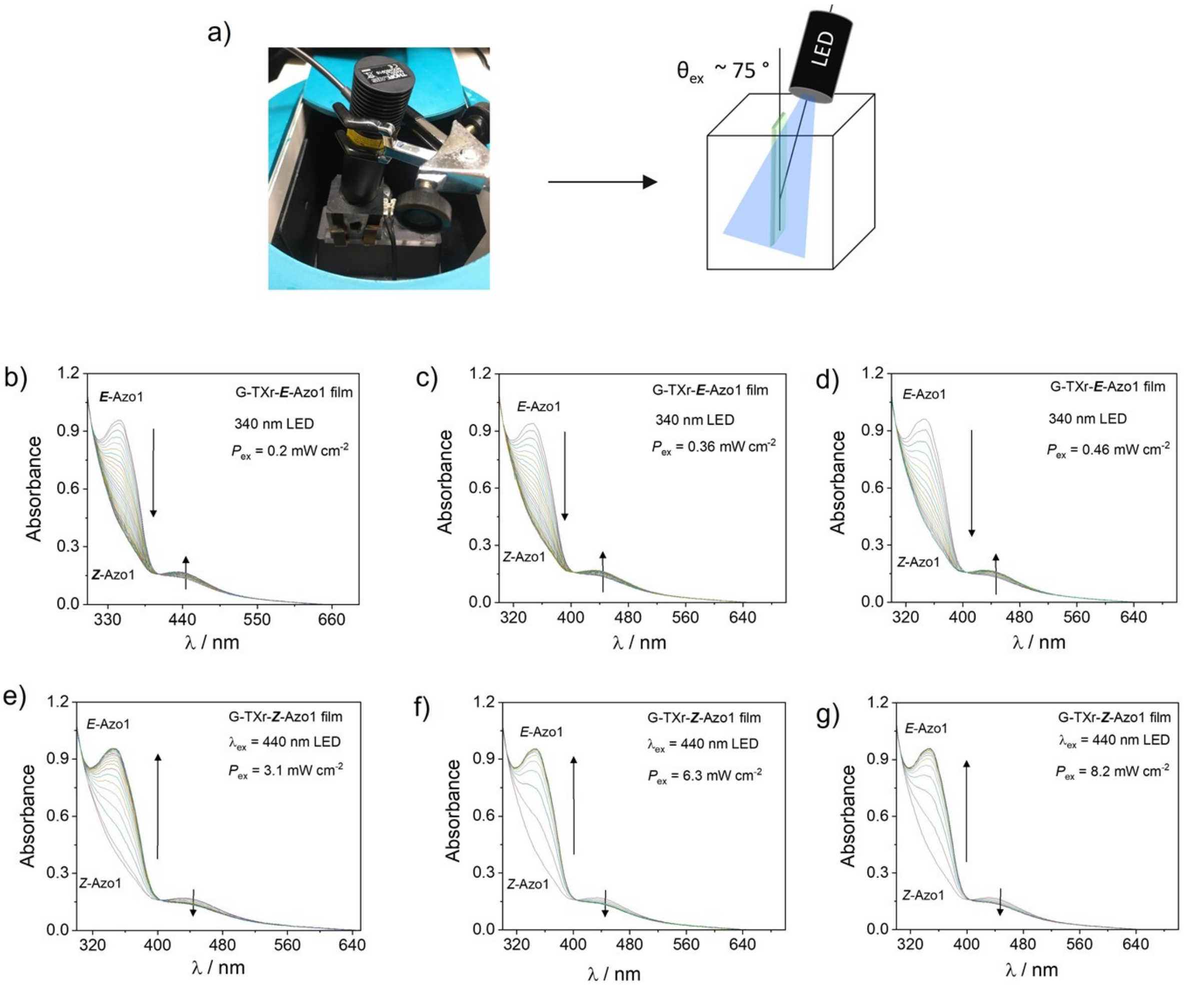

Fig. S10. Kinetics of the photoisomerization in G-TXr-AZO1 film. a) Digital image and model of G-TXr-Azo1 film excitation with 340 and 440 nm LED at ~75 ° angle, b-d) time-dependent absorption spectra of G-TXr-*E*-Azo1 film at different excitation power density of 340 nm LED, and e-f) time-dependent absorption spectra of G-TXr-*Z*-Azo1 film at different excitation power density (P_{ex})of 440 nm LED.

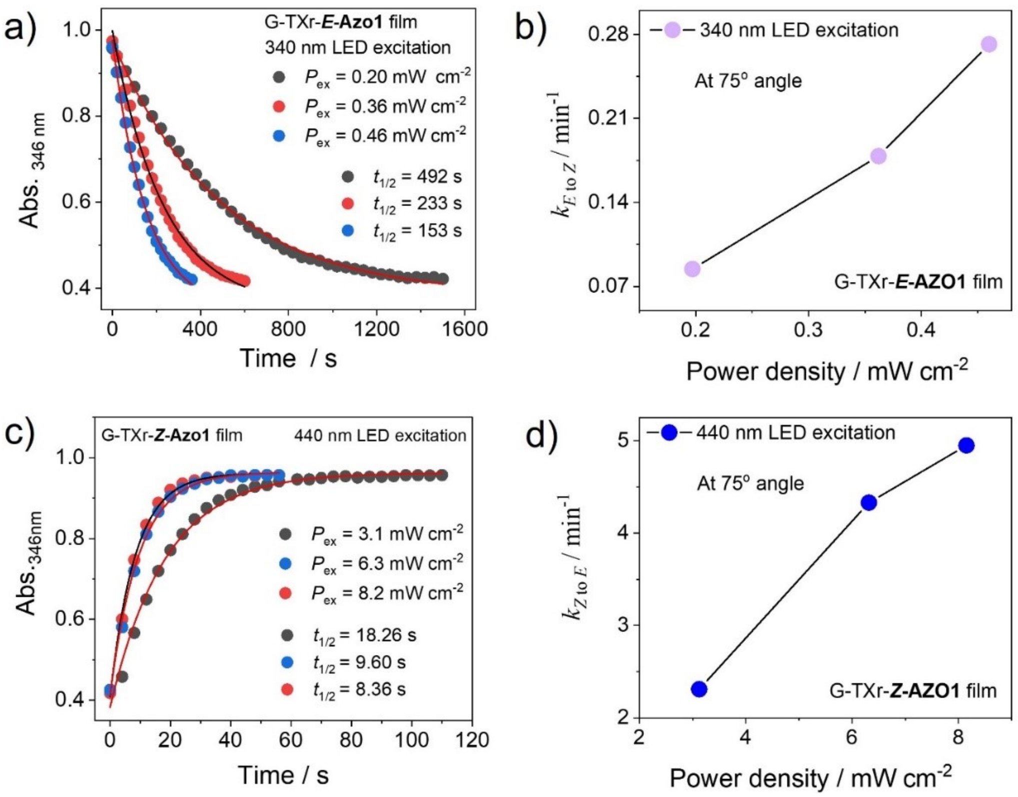

Fig. S11. Kinetics of photoisomerization in G-TXr-Azo1 film. a, c) Absorbance vs time plots of G-TXr-Azo1 film at different excitation power density of 340 nm and 440 nm LED (lines indicating a single exponential fit) and b, d) rate constant of photoisomerization vs. excitation power plots of G-TXr-Azo1 film at different excitation power density of 340 nm and 440 nm LED respectively (lines are just guides to the eye).

Table 3. Rate constants *k* (s^{-1}) of Photoisomerization of Azo1 in the G-TXr-AZO1 film at different excitation powers density (P_{ex}) of 340 and 440 nm LED light.

340 nm LED		440 nm LED	
P_{ex} (mW cm^{-2})	$k_{\text{E to Z}}$ (s^{-1})	P_{ex} (mW cm^{-2})	$k_{\text{Z to E}}$ (s^{-1})
0.20	0.0014	3.1	0.038
0.36	0.003	6.3	0.072
0.46	0.0045	8.2	0.083

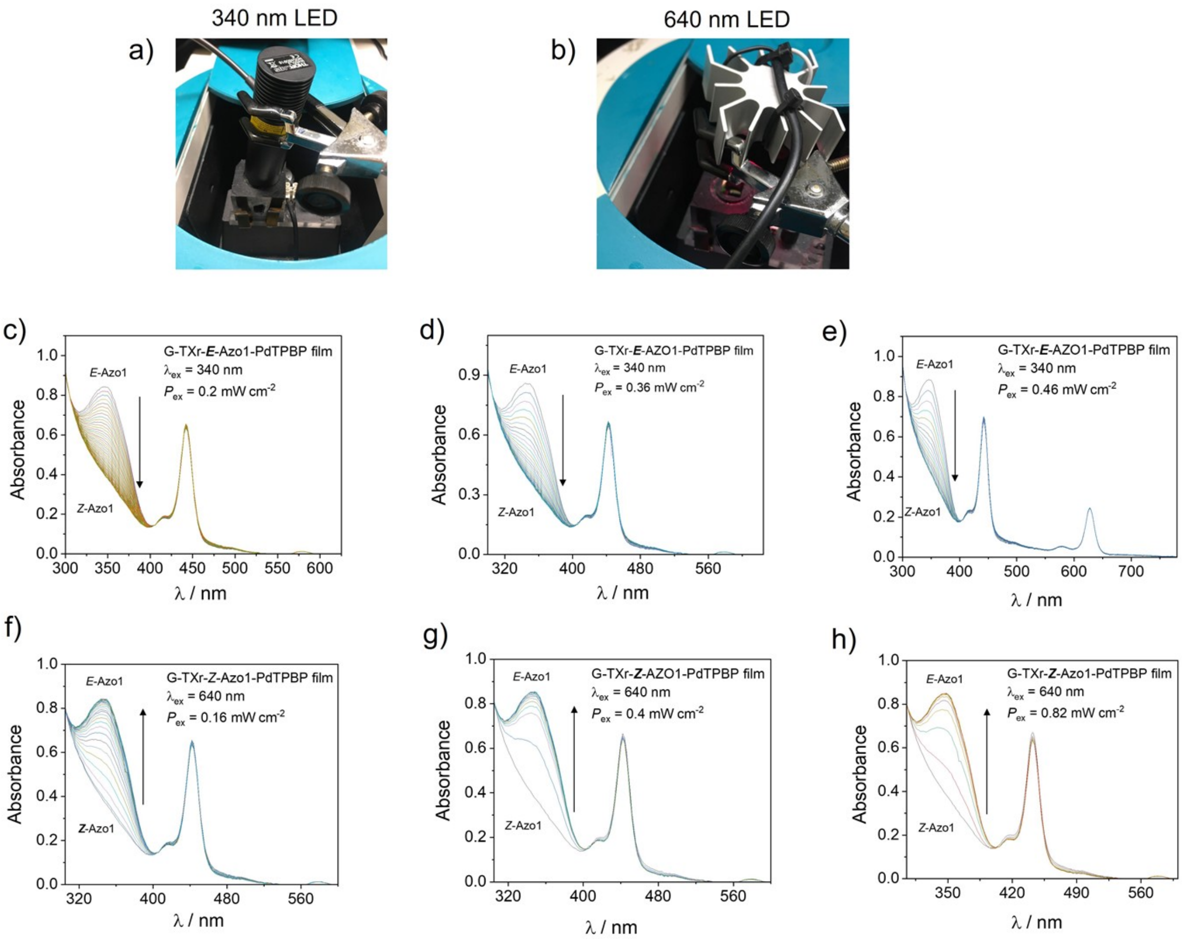

Fig. S12. Kinetics of photoisomerization in G-TXr-Azo1-PdTPBP film. a and b) Digital image of G-TXr-Azo1-PdTPBP film excitation with 340 and 640 nm LED at ~75 ° angle, c-e) time-dependent absorption spectra of G-TXr-*E*-Azo1-PdTPBP film at different excitation power density of 340 nm LED, and e-f) time-dependent absorption spectra of G-TXr-*Z*-Azo1-PdTPBP film at different excitation power density of 640 nm LED.

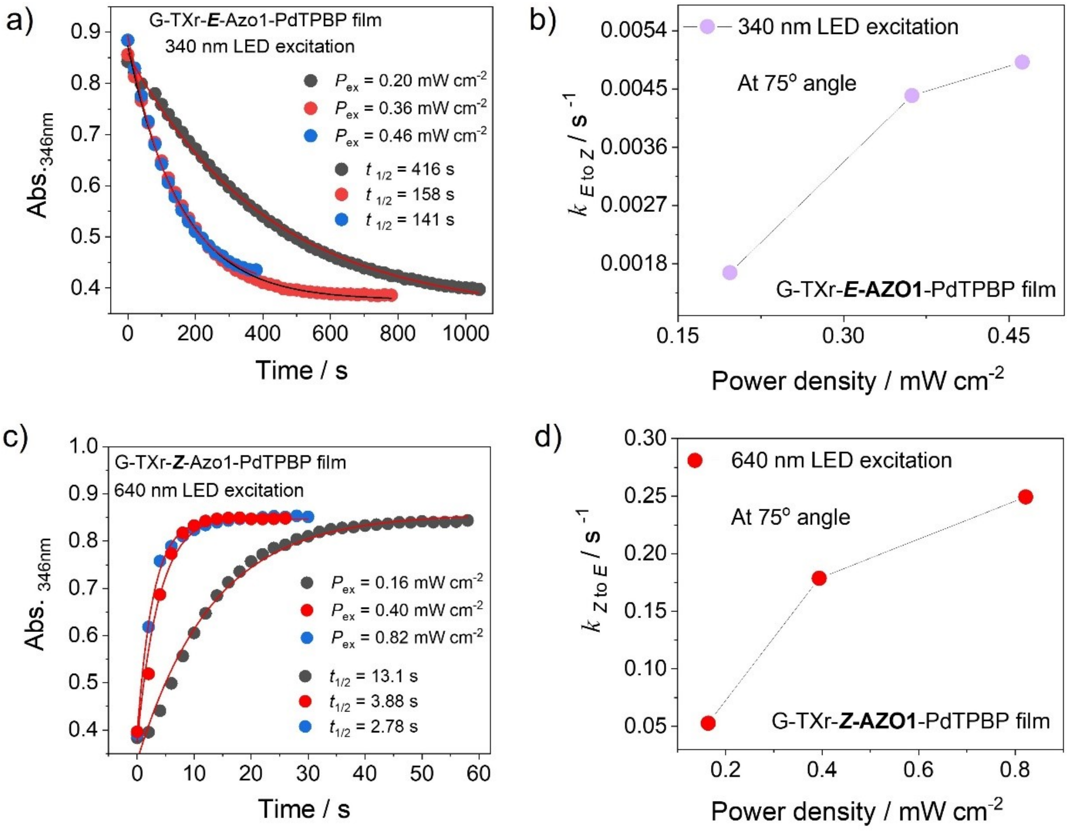

Fig. S13. Kinetics of photoisomerization in G-TXr-Azo1-PdTPBP film. a, c) Absorbance vs. time plots of G-TXr-Azo1-PdTPBP film at different excitation power density of 340 nm and 640 nm LED (lines indicating a single exponential fit), and b, d) rate constant of photoisomerization vs. excitation power density plots of G-TXr-Azo1-PdTPBP film at different excitation power density of 340 nm and 640 nm LED respectively (lines are just guides to the eye).

Table 4. Rate constants k (s^{-1}) of Photoisomerization of Azo1 in the G-TXr-Azo1-PdTPBP film at different excitation power density (P_{ex}) of 340 and 640 nm LED light.

340 nm LED		640 nm LED	
P_{ex} (mW cm^{-2})	$k_{\text{E to Z}}$ (s^{-1})	P_{ex} (mW cm^{-2})	$k_{\text{Z to E}}$ (s^{-1})
0.2	0.0016	0.16	0.053
0.36	0.0044	0.40	0.18
0.46	0.0049	0.82	0.25

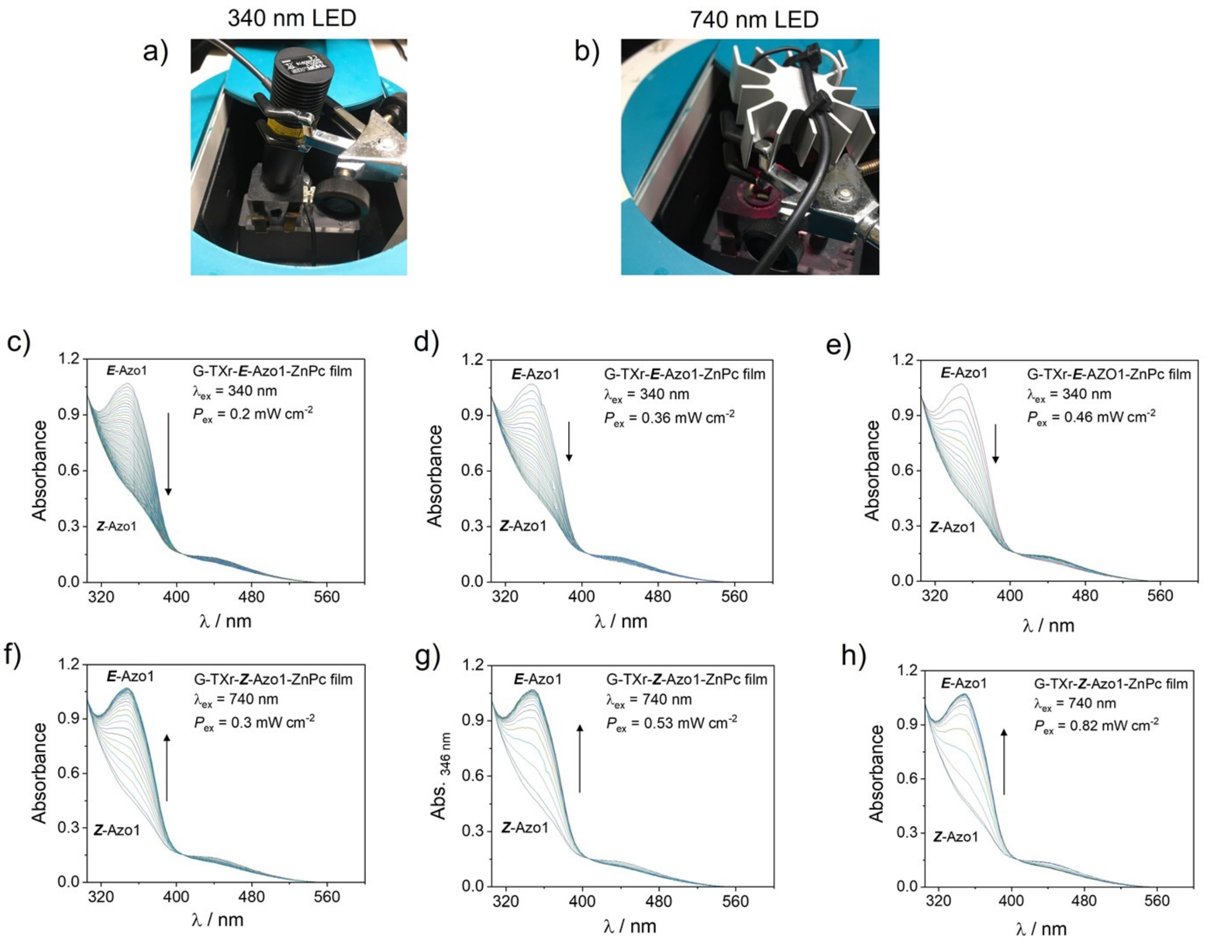

Fig. S14. Kinetics of photoisomerization in G-TXr-Azo1-ZnPc film. a and b) Digital image of G-TXr-Azo1-ZnPc film excitation with 340 and 740 nm LED at ~75 ° angle, c-e) time-dependent absorption spectra of G-TXr-*E*-Azo1-ZnPc film at different excitation power density of 340 nm LED, and e-f) time-dependent absorption spectra of G-TXr-*Z*-Azo1-ZnPc film at different excitation power density of 740 nm LED.

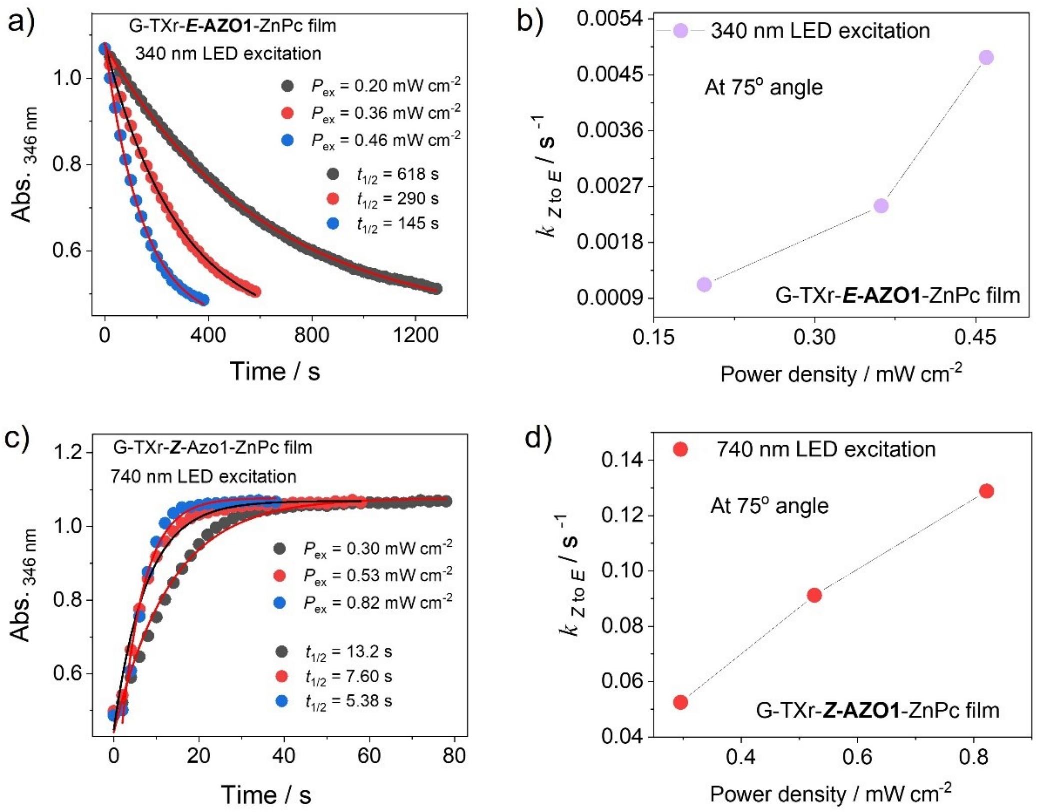

Fig. S15. Kinetics of photoisomerization in G-TXr-Azo1-ZnPc film. a, c) Absorbance vs. time plots of G-TXr-Azo1-ZnPc film at different excitation power density of 340 nm and 740 nm LED (lines indicating a single exponential fit), and b, d) rate constant of photoisomerization vs. excitation power density plots of G-TXr-Azo1-ZnPc film at different excitation power density of 340 nm and 740 nm LED respectively (lines are just guides to the eye).

Table 5. Rate constants, k (s^{-1}) of Photoisomerization of Azo1 in the G-TXr-Azo1-ZnPc film at different excitation power density (P_{ex}) of 340 and 740 nm LED light.

340 nm LED		740 nm LED	
P_{ex} (mW cm^{-2})	$k_{\text{E to Z}}$ (s^{-1})	P_{ex} (mW cm^{-2})	$k_{\text{Z to E}}$ (s^{-1})
0.2	0.0011	0.30	0.052
0.36	0.0024	0.53	0.091
0.46	0.0048	0.82	0.13

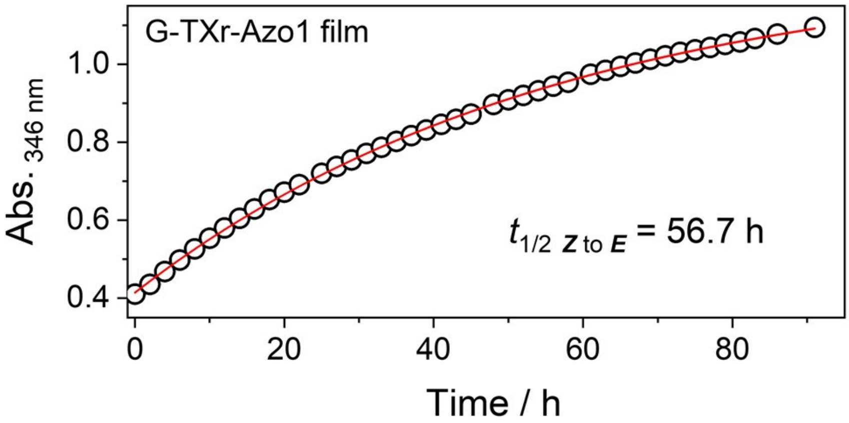

Fig. S16. Kinetics of *Z*-Azo1 to *E*-Azo1 isomerization in G-TXr-Azo1 film at room temperature. a) Absorbance spectrum of G-TXr-***Z***-Azo1 film with time (lines indicating a single exponential fit), and b) absorption vs. time plot of G-TXr-***Z***-Azo1 film.

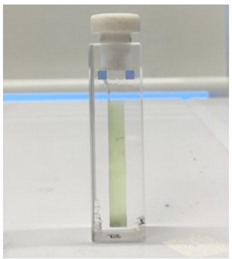

Fig. S17. Digital image of G-TXr-Azo1-PdTPBP or G-TXr-Azo1-ZnPc films fixed inside the quartz cuvette.

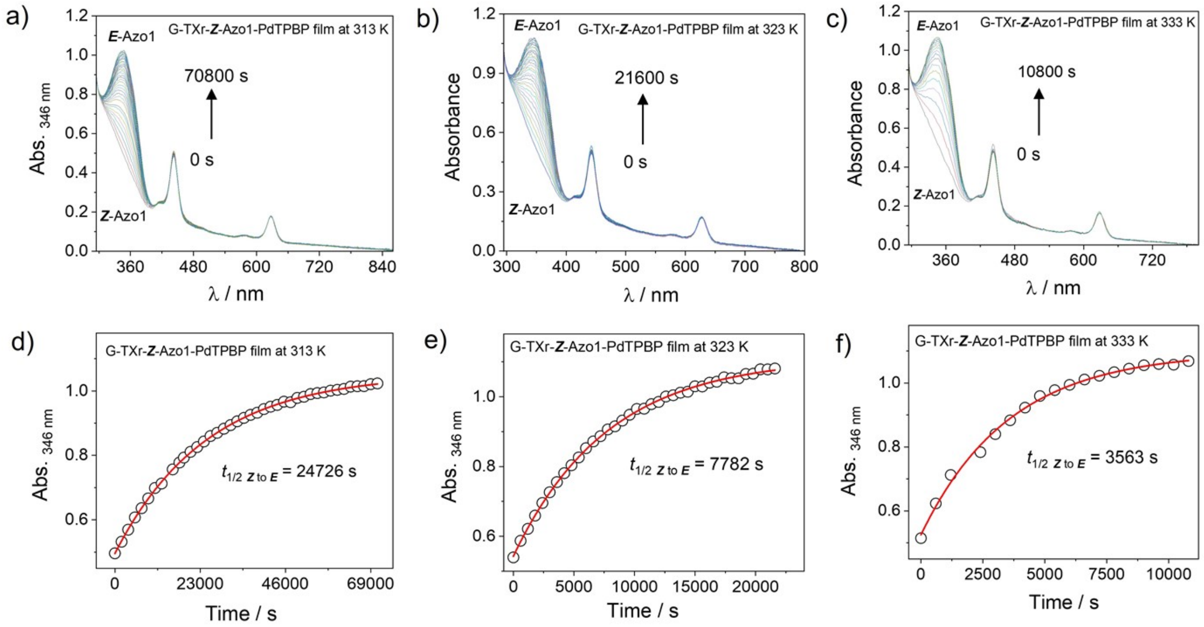

Fig. S18. a to c) Time dependent absorption spectra, and d to f) absorbance vs time plots of G-TXr-Azo1-PdTPBP film at 313 K, 323 K, and 333 K.

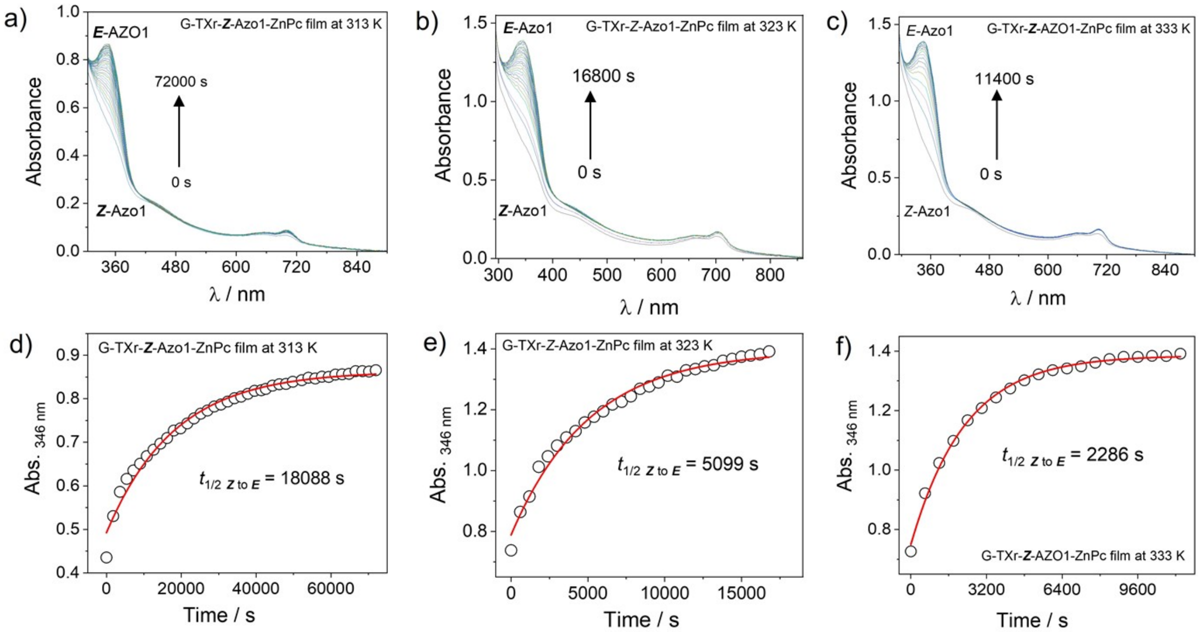

Fig. S19. a to c) Time dependent absorption spectra, and d to f) absorbance vs. time plots of G-TXr-Azo1-ZnPc film at 313 K, 323 K, and 333 K.

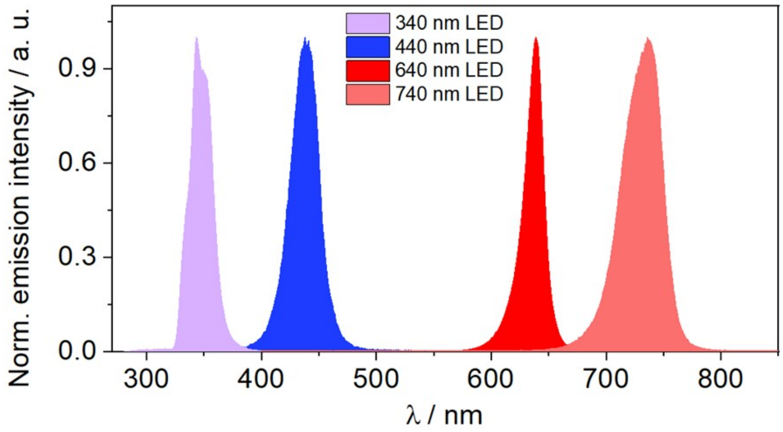

Fig. S20. Emission spectra of LED's used for this work

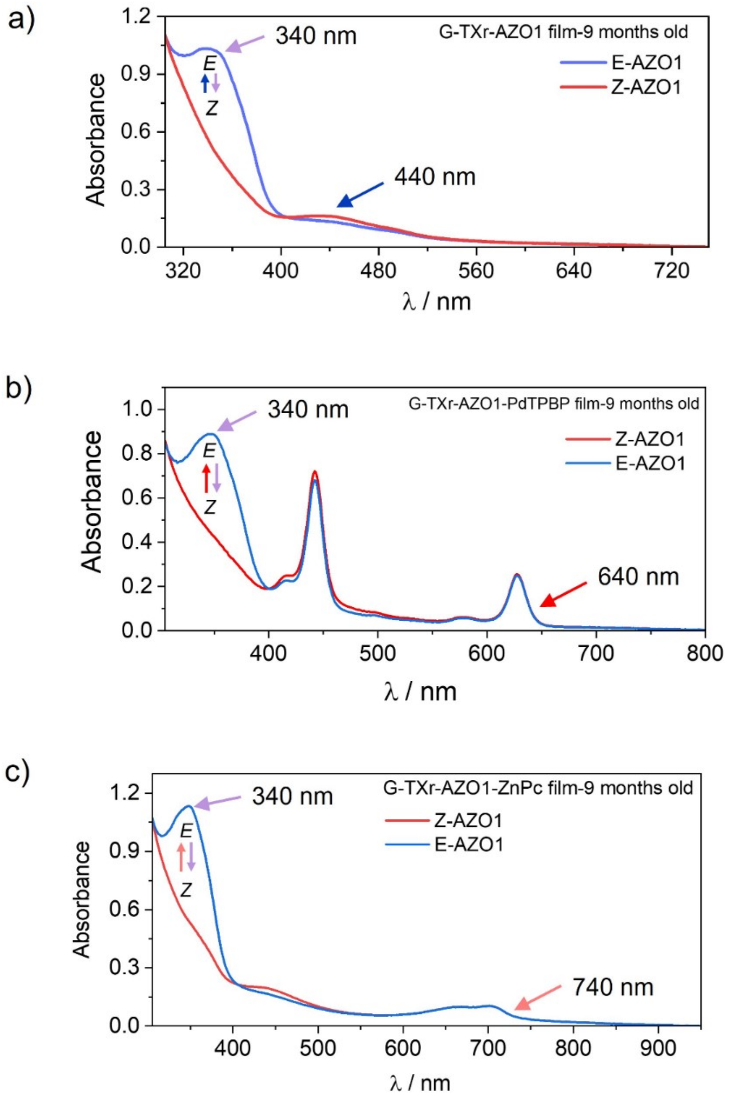

Fig. S21. Absorption spectra of 9 months old a) G-TXr-Azo1 film, b) G-TXr-Azo1-PdTPBP film, and c) G-TXr-Azo1-ZnPc film showing reversible $E \leftrightarrow Z$ photoswitching upon LED's excitation at 90 ° angle. P_{ex} (340 nm LED = 2.8 mW cm^{-2}, 440 nm LED = 27.6 mW cm^{-2}, 640 nm LED = 14.1 mW cm^{-2}, and 740 nm LED = 17.1 mW cm^{-2}.

References.

1. K. Masutani, M,-aki Morikawa, N. Kimizuka, *Chem. Commun*., 2014, **50**, 15803-15806.
2. A. G. Gürek, Özer Bekaroğlu, *J. Chem. Soc. Dalton Trans*. 1994, 1419-1423.
3. L. Gagliardi, G. Orlandi, F. Bernardi, A. Cembran, M. Garavelli, *Theor. Chem. Acc*. 2004, **111**, 363–372
